# Ramadan Fasting and Complications of Metabolic Dysfunction-Associated Steatotic Liver Disease: Impacts on Liver Cirrhosis and Heart Failure

**DOI:** 10.3390/jcm14061841

**Published:** 2025-03-09

**Authors:** Musaab Ahmed

**Affiliations:** 1 College of Medicine, Ajman University, Ajman P.O. Box 346, United Arab Emirates; m.omer@ajman.ac.ae; 2 Center of Medical and Bio-Allied Health Sciences Research, Ajman University, Ajman P.O. Box 346, United Arab Emirates

**Keywords:** Ramadan fasting, metabolic-dysfunction-associated steatotic liver disease, heart failure, insulin resistance, body weight

## Abstract

Background: Metabolic-dysfunction-associated steatotic liver disease (MASLD) and heart failure are two intersecting growing pandemics. Studies have demonstrated a strong association between MASLD and heart failure. Liver cirrhosis is a well-recognized complication of MASLD. This study aimed to summarize the potential effects of Ramadan fasting on MASLD, liver cirrhosis, and heart failure. The author searched the SCOPUS and PubMed databases using specific terms. The literature review focused on research articles published in English from 2000 to 2024. Twenty-two articles were selected for this narrative review. Ramadan fasting reduced serum cholesterol serum levels, improved symptoms of heart failure and reduced anthropometric measurements. However, it increased ascitic fluid production and plasma bilirubin levels and might increase the risk of hepatic encephalopathy and upper gastrointestinal haemorrhage in liver cirrhosis. Ramadan fasting might improve symptoms of heart failure and might decrease the risk of heart failure in patients with MASLD. Further research studies are needed to confirm the efficacy and evaluate the safety of Ramadan fasting in patients with heart failure and liver cirrhosis.

## 1. Introduction

Metabolic-dysfunction-associated steatotic liver disease (MASLD) and heart failure (HF) are becoming significant clinical and public health issues worldwide [1,2]. Individuals diagnosed with MASLD have heightened vulnerability to mortality resulting from cardiovascular and hepatic consequences. MASLD has become a prominent indication for liver transplantation [3]. MASLD is a comprehensive term that encompasses a number of disorders, from simple hepatic fat accumulation to more severe conditions such as steatosis with hepatitis, cirrhosis fibrosis, and hepatocellular carcinoma, all occurring in the absence of significant use of alcohol [4]. MASLD is characterized by the buildup of fat in the liver, including above 5% of the liver tissue, without resulting in cellular damage. MASH is a disorder marked by inflammation and hepatocellular apoptosis, accompanied by the buildup of lipids [5]. Most patients diagnosed with MASLD display insulin resistance and obesity, which substantially contributes to the onset of metabolic syndrome (MetS) [6,7]. MASLD is considered the liver manifestation of MetS, characterized by a constellation of metabolic disorders including hypertriglyceridemia, central obesity, hypertension, hyperglycemia, and decreased high-density lipoprotein levels. Both MetS and MASLD increase the risk of type 2 diabetes, stroke, and cardiovascular disease [8]. The global prevalence of MASLD is approximately 25% [9]. At present, there is no single pharmacological treatment for MASLD. The drug resmetirom shows promising results in the management of NASH and liver fibrosis [10]. One of the important hepatic complications of MASLD is liver cirrhosis. Cirrhosis has emerged as a critical public health concern and a substantial contributor to morbidity and mortality [11,12]. Compelling evidence demonstrates that the clinical burden of MASLD extends beyond liver-related complications and negatively impacts various extrahepatic systems, including the cardiovascular system [13]. Specifically, MASLD is linked to a significantly elevated risk of experiencing significant detrimental cardiovascular events which are the primary cause of mortality in individuals with MASLD [14]. Additionally, it is correlated with an increased risk of cardiac arrhythmias, particularly atrial fibrillation, and myocardial remodeling, which might facilitate the onset of new heart failure [15]. There is an increasing interest in dietary intervention as a possible treatment for diseases [16,17]. Intermittent fasting (IF) has attained significant popularity as a new approach to reduce body weight [18]. Studies suggested that the health benefits of IF extend beyond weight reduction [19]. Ramadan fasting has numerous health benefits including weight reduction, reduction in oxidative stress, and enhancement in blood glucose levels [20]. Here, we review pathogenesis MASLD and summarize the possible effects of Ramadan intermittent fasting on MASLD, liver cirrhosis, and heart failure.

## 2. Methods

**Figure 1:**
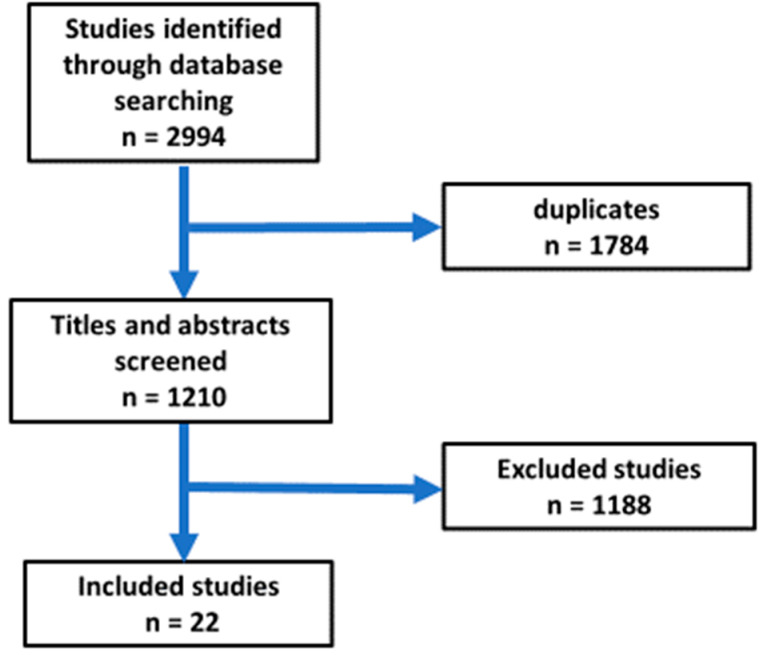
Flow chart for the selection of articles included in the review.

This research was performed as a narrative review. The author conducted a search of the SCOPUS and PubMed databases utilizing the terms: nonalcoholic fatty liver, heart failure, liver cirrhosis, and Ramadan fasting. The author performed a search utilizing a combination of the following terms: [pathophysiology AND Nonalcoholic fatty liver] OR [Ramadan Fasting AND nonalcoholic fatty liver OR heart failure OR liver cirrhosis AND clinical trials]. The literature search focused on research articles published in English between 1 January 2000, and 30 August 2024, followed by the screening of abstracts and articles. Articles pertinent to the subject were systematically examined, and information concerning Ramadan intermittent fasting, study duration, participant count, and impacts on heart failure, liver cirrhosis and nonalcoholic fatty liver was extracted. The reference list contained pertinent citations. The initial search yielded a total of 2994 publications. A total of 22 articles were selected for the review (see Figure 1). The criteria employed for article selection were as follows.

The review included randomized controlled trials, observational studies, experimental studies, and review articles. Only publications authored in English were considered eligible. Studies concerning Ramadan intermittent fasting, nonalcoholic fatty liver, heart failure and liver cirrhosis were included.

Exclusion criteria included the removal of commentaries, letters to the editor, news articles, notes, case reports, books, short surveys, theses, opinions, conference abstracts, repeated studies, and publications that were not written in English.

All retrieved papers were imported into EndNote to eliminate duplicates. Subsequently, abstracts and titles were screened, and article selection was conducted based on the established eligibility criteria. Figure 1 illustrates the flow chart for the selection of publications.

## 3. Pathophysiology of MASLD

The exact pathogenic mechanism of MASLD is intricate and remains incompletely elucidated. The syndrome is primarily characterized by the accumulation of free fatty acids and triglycerides predominantly resulting from insulin resistance and obesity [21]. Insulin resistance has a significant influence on the progression of endoplasmic reticulum stress, oxidative stress, and proinflammatory cytokines. Hepatic steatosis results in hepatic de novo lipogenesis, decreased lipolysis in adipose tissue, and an elevation of liver fatty acids [22]. Furthermore, insulin resistance results in alterations in the synthesis and secretion of inflammatory cytokines and adipokines [23]. The accumulation of triglycerides in the liver enhances the formation of reactive oxygen species, which subsequently causes mitochondrial dysfunction and endoplasmic reticulum stress [24]. The gut microbiota plays a significant role in the development of MASLD. The gut microbiota influences elimination and absorption of nutrient in the liver, along with hepatic inflammation. This occurs as they supply toll-like receptor ligands that activate the liver to generate increased levels of proinflammatory cytokines. Probiotics have been proposed as a possible intervention for MASH through the modification of intestinal bacterial composition [25].

Hypertension, hyperlipidemia, insulin resistance, obesity, diabetes mellitus, and hyperglycemia are complications that may develop in individuals following a liver transplant. Some metabolic abnormalities may partially arise from the administration of drugs like corticosteroids and sirolimus following liver transplantation [26].

Post-liver transplant features encompass MetS characteristics, with MASLD representing the hepatic manifestation. Consequently, individuals can experience a relapse or emergence of new cases of MASLD or MASH following a liver transplant [27,28]. The rate of incidence of MetS following liver transplantation is about 50–60% [29]. A study involving one hundred seventy patients with liver transplants over a duration of 2 years indicated that MetS was present in one-third of these patients [30]. The development of MASLD following a liver transplant may contribute to the increased cardiovascular mortality [31].

## 4. Relationship Between MASLD and Heart Failure

Individuals with MASLD frequently display characteristics typical of MetS and associated cardiovascular disease risk factors [32,33]. MASLD exhibits a significant correlation with metabolic syndrome and obesity and is considered the liver manifestation of MetS. Dyslipidemia, type 2 diabetes, and obesity are identified as major risk factors for MASLD [34]. The precise pathophysiologic mechanisms linking MASLD and heart failure (HF) are not yet completely understood; however, metabolic stress induced by higher serum glucose and free fatty acids, alongside dysregulated insulin signaling, compromised mitochondrial respiration, and the generation of reactive oxygen species, is believed to diminish ATP production. This reduction results in myocellular hypertrophy, compromised myocardial contraction, and cardiac fibrosis, ultimately culminating in heart failure [35]. Research has shown a higher prevalence of heart failure in individuals with MASLD, regardless of diabetes status. Cardiometabolic risk factors of MetS are considered risk factors for both MASLD and heart failure [36,37]. Consequently, MASLD is frequently associated with lifestyle factors, and evidence suggests that lifestyle modifications may reduce transaminase levels, thereby potentially improving MASLD [38]. A research study on individuals with type 2 diabetes mellitus indicated that the incidence of peripheral vascular diseases, cerebrovascular diseases, and coronary diseases was elevated in those with MASLD [39]. Numerous research studies examined the relationship between cardiovascular disease and MASLD. These investigations have consistently demonstrated that cardiovascular disease presents a substantial and imminent threat [40].

## 5. MASLD as an Independent Risk Factor for Heart Failure

In 2023, a thorough meta-analysis included eleven observational cohort studies comprising over eleven million middle-aged subjects from various countries, documenting over ninety-eight thousand instances of new-onset heart failure over a median follow-up period of ten years [41]. The meta-analysis determined that the existence of MASLD (identified through blood biomarkers/scores, ICD-10 codes, imaging modalities, or liver histology) was significantly correlated with more than one-fold increased risk of new-onset heart failure. This risk persisted as substantial even after controlling for sex, age, ethnicity, type 2 diabetes mellitus, hypertension, adiposity metrics, and other cardiometabolic risk factors. The extent of this risk remained constant regardless of stratification by study nation, follow-up period, modality of heart failure diagnosis, or methodologies employed for identifying nonalcoholic fatty liver disease. The chance of developing incident heart failure notably escalated with the increasing severity of MASLD, particularly at advanced fibrosis stages [41]. Recent longitudinal studies corroborate these observations, indicating that an elevated fibrosis-4 (FIB-4) index or other non-invasive liver fibrosis scores correlate with an increased risk of patient hospitalization for heart failure in a substantial real-world cohort of patients with diagnosed NASH or NAFLD [42].

In 2021, Lee et al. utilized a national health screening database comprising approximately nine million middle-aged Koreans, monitored for a median duration of 10.1 years. Their findings indicated that MASLD, defined by a fatty liver index (FLI) of 30 or greater, was significantly correlated with a greater risk of incident heart failure events, with an adjusted hazard ratio of 1.61 and a 95% confidence interval of 1.55 to 1.67. This association remained unaffected by sex, age, household income, and residential area [43].

Simon et al. in a Swedish cohort study involving 10,422 adults with biopsy-confirmed MASLD and nearly 55,000 matched control subjects, investigated the risk of incident major cardiovascular events, including heart failure, over a median follow-up duration of 13.6 years, in relation to the histological severity and presence of NAFLD. The authors observed that, in comparison to matched population controls, NAFLD patients exhibited a significantly elevated incidence of heart failure after controlling for common cardiometabolic risk factors. The incidence rates of heart failure events escalated with the severity of MASLD peaking in cirrhotic and non-cirrhotic fibrosis [44].

Simon et al. investigated the relationship between MASLD and the risk of new-onset heart failure in a national cohort study in Sweden involving 699 obese children and young adults aged 25 years or younger, all with histologically confirmed NAFLD, alongside 3353 control subjects matched for sex age, county, and calendar year for a median follow-up period of 16.6 years. The authors discovered that young patients with NAFLD exhibited significantly higher incidence rates of congestive heart failure compared to matched population controls (adjusted hazard ratio: 3.89; 95% confidence interval: 1.20–12.6), with an even greater increase associated with nonalcoholic steatohepatitis (NASH) [45]. Further research studies should be conducted to elucidate cardiovascular risk in young adults and obese children and with MASLD.

## 6. Ramadan Intermittent Fasting

Intermittent fasting (IF) is an intervention that encompasses dietary alterations and behavioral changes, characterized by alternating periods of fasting and controlled feeding. The length of fasting varies between different regimens. Ramadan fasting is a religious observance for Muslims, occurring for one month each lunar year [46]. Ramadan intermittent fasting is a distinctive form of intermittent fasting during which Muslims are forbidden to consume food, drink, smoke or take medicine between dusk and sunrise during the month of Ramadan [47,48]. Fasting throughout the month of Ramadan is mandatory for all physically capable adult Muslims. The duration of the daily fast during the month of Ramadan differs throughout countries and fluctuates annually. The duration may vary from a few hours to more than twenty hours, commencing at sunrise and concluding at nightfall [48]. As a result, extended periods of daily fasting are seen during the summer. During Ramadan, Muslims keeping the fast must abstain from all forms of oral consumption, including eating, drinking, and smoking, from before sunrise until after sunset. Most of individuals observing Ramadan consume two meals daily: Suhoor, taken before dawn to initiate the fast, and Iftar, consumed after dusk to conclude the fasting [49]. Ramadan presents difficulties for individuals with type 2 diabetes mellitus who opt to fast. Ensuring adequate glycemic control with consistent monitoring during fasting is essential [48,50]. Ramadan fasting possesses numerous health effects including weight reduction, reduction in inflammatory markers and oxidative stress, and enhancement in blood glucose levels [20,51].

## 7. Impacts of Ramadan Fasting on Metabolic-Dysfunction-Associated Steatotic Liver Disease

Limited research has assessed the effects of Ramadan fasting on MASLD (Table 1). Ramadan fasting may serve as a potent dietary intervention for MASLD. High-quality clinical studies are needed to confirm the efficacy of Ramadan fasting as a dietary intervention for MASLD.

## 8. Ramadan Fasting and Heart Failure

Only seven studies assessed the impact of Ramadan on heart failure. An observational study conducted by Salam et al. in seven countries in the Middle East included 4157 patients with acute heart failure and showed that patients admitted during Ramadan (307 patients) exhibited a significantly lower incidence of signs and symptoms of volume overload in comparison to those hospitalized in the other months. cholesterol levels were significantly reduced, and atrial arrhythmias were notably less frequent during Ramadan. Furthermore, hospitalization of patients in the month of Ramadan was not independently linked to increased immediate or one-year mortality [58]. Another observational study conducted by Abazid et al. in Saudi Arabia included 249 patients and showed that Ramadan fasting is generally deemed safe for most of patients with chronic heart failure. Non-adherence to medication and dietary guidelines is significantly correlated with decompensated heart failure during the month of Ramadan [59]. In the same direction, a study by Alam et al. in Pakistan included 938 patients and reported that fasting during Ramadan improved the symptoms and quality life of chronic heart failure patients. The authors suggested that heart-failure patients should be encouraged to fast during Ramadan [60]. Al Suwaidi et al. in Qatar included 20,856 patients with congestive heart during a ten-year period from January 1991 to December 2001 to evaluate the effect of Ramadan fasting on the number of hospitalizations for congestive heart failure. The study indicated no significant difference in the number of patient hospitalizations for congestive heart failure during Ramadan fasting compared to during other months [61]. Another study by Al Suwaidi et al. in Kuwait, United Arab Emirates, Qatar, and Bahrain included 465 patients to investigate the effect of fasting during Ramadan on patients with heart disease including congestive heart failure. The study found that the effect of Ramadan fasting on stable patients with cardiac disease is negligible. Patients with stable cardiac disease are generally able to fast [62]. Similarly, Chamsi-Pasha and Ahmed’s study in Saudi Arabia included 86 patients to investigate the effect of Ramadan fasting on patients with heart disease including congestive heart failure found that the impact of Ramadan fasting on stable patients with cardiac disease is negligible. Most of the patients with stable cardiac disease can observe fasting during Ramadan without notable adverse effects [63]. Alaarag et al. in Saudi Arabia involving 158 patients demonstrated that fasting throughout Ramadan may be safe for low-risk patients with chronic heart failure and reduced ejection fraction when monitored by a healthcare practitioner. Patients with chronic heart failure and reduced ejection fraction who have a history of coronary revascularization or chronic renal disease may experience a greater prevalence of adverse effects following Ramadan fasting [64]. Further research studies are needed to evaluate the impacts of Ramadan fasting on heart failure and to evaluate the safety of Ramadan intermittent fasting for patients with heart failure.

## 9. Impacts of Ramadan Fasting on Blood Pressure

Six studies reported the impacts of Ramadan fasting on blood pressure. An observational study conducted by Nematy et al. on 82 Muslim patients with one cardiovascular risk factor who fasted during Ramadan found a significant reduction in systolic blood pressure, while the change in diastolic blood pressure was not significant [65]. Farag et al., involving one hundred twenty patients with hypertension, demonstrated that fasting during Ramadan produced a significant decrease in blood pressure [66]. Bener et al., involving 1246 individual with diabetes, demonstrated a significant reduction in systolic and diastolic blood pressure [67]. Ouselati et al., involving 52 individuals with type 2 diabetes mellitus, demonstrated that Ramadan intermittent fasting produced no significant changes in blood pressure [68]. Similarly, Zairi et al. in Tunisia, involving 60 hypertensive patients, demonstrated no significant changes in diastolic and systolic blood pressure [69]. Another study by Norouzy et al. in Saudi Arabia involving 18 patients found that there were no significant differences in diastolic and systolic blood pressures between the hypertensive and normotensive patients during the month of Ramadan and one month after it [70].

Intermittent fasting has been observed to possess the potential to reduce blood pressure, thereby potentially enhancing cardiovascular disease mortality. The proposed mechanisms for the reduction in blood pressure by intermittent fasting are activation of the parasympathetic nervous system through an increase in the discharge of brainstem cholinergic neurons [71], reduction in the activity of the renin angiotensin aldosterone system, and manipulation of gut microbiota. Vascular potassium channels play an important role in vasodilation of blood vessels by causing relaxation of vascular smooth muscle [72,73]. Obesity alters the sensitivity, expression, and function of vascular potassium channels causing dysfunction of smooth muscle [74]. As intermittent fasting can cause a reduction in body weight, it can modulate the activity of potassium channels, counteract vascular dysfunction and cause a reduction in blood pressure.

## 10. Ramadan Fasting and Endothelial Dysfunction

Endothelial dysfunction plays a significant role in the pathogenesis of heart failure, since recurrent instances of microvascular dysfunction may lead to myocardial stunning and remodeling of the ventricles and might be associated with hospitalizations due to heart failure [75]. Four studies evaluated the impacts of Ramadan fasting on endothelial dysfunction. A study conducted by Demirci and Özkan in Turkey investigated the effect of Ramadan fasting on endothelial dysfunction in hypertensive patients. Flow-mediated dilatation was assessed before and after fasting during Ramadan. The study showed a significant improvement in flow-mediated dilatation after Ramadan. This improvement might be attributed to decreased cortisol and CRP levels after Ramadan [76]. Another study was conducted by Tahapary et al. to evaluate the effect of Ramadan fasting on the biological marker of endothelial dysfunction, ICAM-1, in diabetic and non-diabetic individuals. The study showed a significant reduction in the level of ICAM-1 in both diabetic and non-diabetic individuals [77]. Goser et al. included 67 patients with slow coronary flow in a retrospective study to assess the effect of Ramadan fasting on endothelial dysfunction using TIMI frame count. The study showed that Ramadan fasting and other lifestyle changes during Ramadan could improve endothelial dysfunctions [78]. A study by Yousefi et al. in Iran include 21 patients and showed that the levels of nitric oxide (NO) were significantly higher in patients after Ramadan fasting (85.1 ± 11.54 vs. 75.8 ± 10.7 μmol/L; *p* < 0.05)). Post-Ramadan levels of asymmetric dimethylarginine (ADMA) reduced significantly (*p* < 0.05). In addition, the levels of vascular endothelial growth factor (VEGF) increased, and the levels of malondialdehyde (MDA) decreased during Ramadan fasting, but these changes were not statistically significant [79]. Ramadan fasting might improve endothelial dysfunction and increased bioavailability of nitric oxide.

## 11. Ramadan Fasting and Autophagy

**Figure 2:**
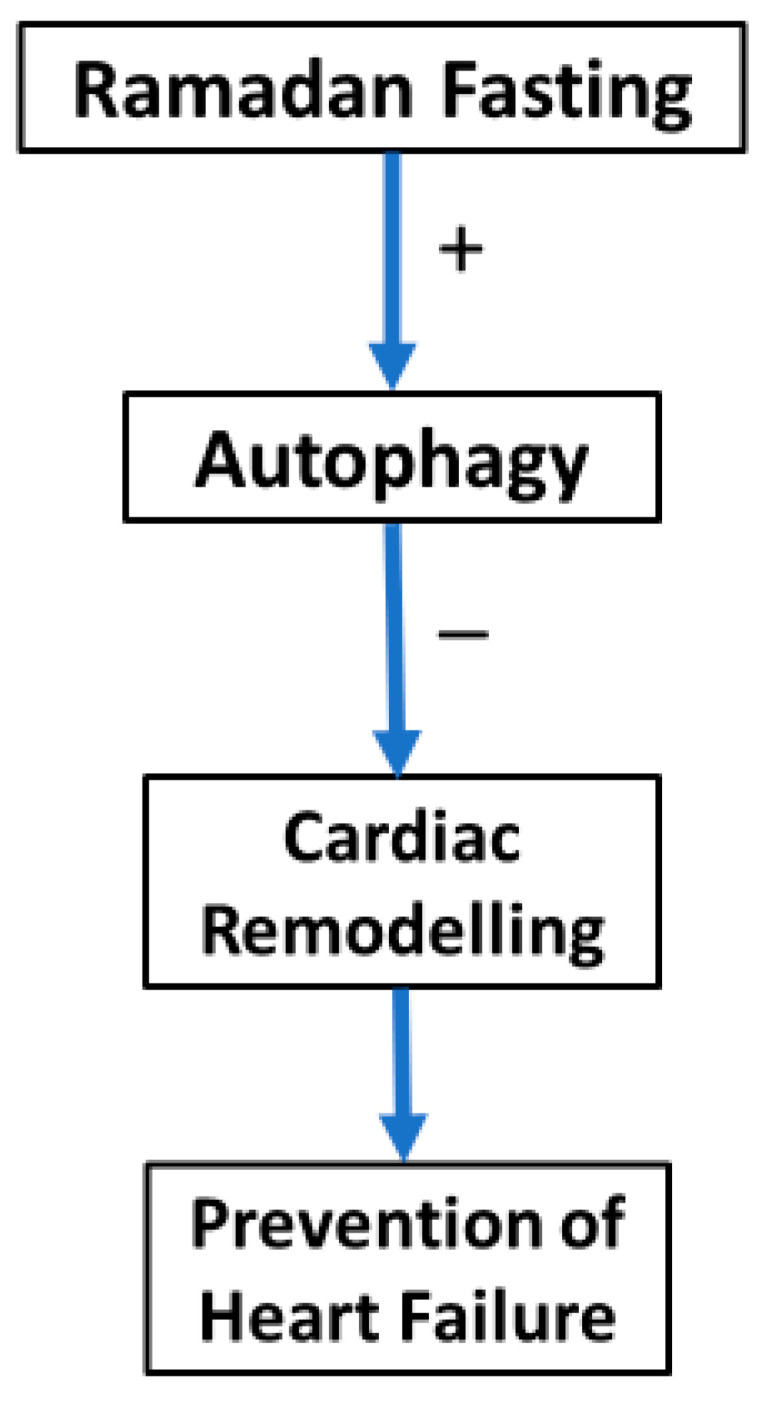
Relationship between Ramadan fasting, autophagy, and heart failure.

Intermittent fasting stimulates autophagy by the activation of lysosomes, which might contribute to a reduction in myocardial remodelling and an improvement in cardiac performance [80]. Research studies indicated that autophagy stimulation inhibits cardiac hypertrophy and ameliorates diastolic dysfunction caused by aging and various stressors [81,82]. Bou Malhab et al. 2024 in United Arab Emirates included 51 participants with overweight and obesity and demonstrated that Ramadan is linked to the elevation in autophagy gene expressions (LAMP2, LC3B, ATG5, and ATG4D) in individuals with overweight or obesity, which may partially elucidate its beneficial short-term metabolic and health-enhancing effects on early aging-related markers. Consequently, Ramadan fasting may confer a preventive effect against early indicators of metabolic disorders in individuals with overweight or obesity [83] (Figure 2).

## 12. Ramadan Fasting and Liver Cirrhosis

Liver cirrhosis can arise from various etiologies, with the most significant being MASLD. Liver cirrhosis is a gradual degenerative condition characterized by the replacement of normal liver tissue with disorganized tissue exhibiting varying degrees of fibrosis, leading to functional impairment of the liver, and can be associated with portal hypertension [84,85]. The worldwide prevalence of liver cirrhosis ranges from 4.5% to 9%. Management of cirrhosis and its complications incurs substantial costs and imposes a significant strain on society [46]. Liver cirrhosis may further aggravate with the onset of hepatocellular carcinoma presenting an additional obstacle in the treatment of afflicted individuals [86].

A few studies investigated the effect of Ramadan fasting ion liver cirrhosis. An observational study by Elnadry et al. in Egypt involving 202 patients with chronic liver disease investigated the effect of Ramadan fasting on chronic liver disease and demonstrated that fasting cirrhotic patients had a high deterioration to Child−Pugh class C at the end of Ramadan [87].

Mohamed et al.’s observational study in Egypt included 40 patients with liver cirrhosis to evaluate the effects of Ramadan fasting on liver cirrhosis and portal blood flow. Cirrhotic patients exhibited notable short-term alterations in portal blood flow. Seven patients experienced problems, including two instances of variceal hemorrhage. Due to noted impairments in liver function, patients classified as Child−Pugh class C should refrain from fasting [88].

Emara et al. in a review demonstrated that patients with Child−Pugh class A cirrhosis may fast during Ramadan, especially if they comply with NAFLD recommendations, subject to previous evaluations and careful monitoring during the month. Patients with cirrhosis categorized as Child−Pugh class B and C should avoid fasting. The likelihood of decompensation is elevated [89]. Al-Jafar et al. conducted the LORANS study and a meta-analysis and showed that Ramadan fasting correlated with decreases in anthropometric measurements in patients with cirrhosis. These alterations commenced in the second week of Ramadan and subsided three weeks post-Ramadan [90]. Another observational study by Mohamed et al. included 72 subjects and demonstrated that patients with liver cirrhosis exhibited alterations in liver function and portal hemodynamics, regardless of fasting status, with more significant variations observed in the portal vein congestion index, Model for End-Stage Liver Disease (MELD) score, and serum albumin compared to healthy individuals [91].

Emara et al., in a review, showed that patients with liver cirrhosis might develop increased ascitic fluid production, new ascites, and elevated plasma bilirubin levels post-Ramadan and might frequently experience hepatic encephalopathy and abrupt upper gastrointestinal haemorrhage. The incidence of these consequences was elevated in individuals with Child−Pugh class B and C cirrhosis, with several fatalities attributed to Ramadan fasting [46]. Patients with cirrhosis classified as Child−Pugh class B and C should refrain from fasting throughout Ramadan, although those classified as Child−Pugh class A may fast if they adhere to specific precautions.

## 13. Study Strengths and Limitations

The caliber of the assessed research papers is a notable strength of this assessment. This study was performed as a narrative review rather than a systematic review and offered current information on the subject. The objective of this narrative review was to provide readers with a comprehensive assessment of the topic while also offering a concise update on the subject matter. This review has some limitations. The author used free search engines for literature retrieval, encountering limitations in completely accessing some publications. The author included only papers published in English; nonetheless, given the volume of studies included in the review, the few omitted studies would have a negligible influence on the study’s outcomes.

## 14. Conclusions

Metabolic-dysfunction-associated steatotic liver disease and heart failure are two growing worldwide problems. MASLD is strongly associated with heart failure. Ramadan intermittent fasting produced improvements in the cardiometabolic risk factors associated with MASLD and heart failure. Moreover, Ramadan fasting has the potential to improve MASLD. Moreover, Ramadan intermittent fasting improved symptoms of heart failure, and patients with heart failure can be encouraged to fast during Ramadan. Furthermore, Ramadan fasting might decrease the risk of heart failure in patients with MASLD. Patients with cirrhosis classified as Child−Pugh class B and C should refrain from fasting throughout Ramadan, although those classified as Child−Pugh A may fast if they adhere to specific precautions (Table 2). Large randomized controlled trials are needed to confirm the efficacy and evaluate the safety of Ramadan intermittent fasting in patients with liver cirrhosis and heart failure.

## Figures and Tables

**Table 1:** Summary of published studies on the impact of Ramadan fasting on MASLD.

Authors	Main Finding	Reference
Alasmari et al. 2024	Ramadan fasting resulted in a significant decrease in body weight, serum levels of cholesterol, LDL, triglycerides, liver histology, and the enzymes aspartate transaminase and alanine transaminase in experimental animals	[52]
Mari et al. 2021	Ramadan fasting resulted in improvements in insulin sensitivity, body weight, inflammatory markers, and noninvasive measures for assessing the severity of NASH (the body mass index reduced from 36.7 ± 7.1 to 34.5 ± 6.8 following Ramadan fasting (*p* < 0.003); the NAFLD fibrosis score decreased from 0.45 ± 0.25 to 0.23 ± 0.21 (*p* < 0.005); the FIB4 score declined from 1.93 ± 0.76 to 1.34 ± 0.871 (*p* < 0.005); C-reactive protein reduced from 14.2 ± 7.1 to 7.18 ± 6.45 (*p* < 0.005)) in 155 patients with NASH.	[53]
Ebrahimi et al. 2020	Ramadan fasting resulted in a significant reduction in anthropometric indices total cholesterol (*p* = 0.02), as well as reductions in the Atherogenic Index of Plasma (AIP) and the Visceral Adiposity Index (VAI) among the patients (*p* = 0.79 and *p* = 0.65 for the AIP and the VAI, respectively). It also led to improvements in concentrations of liver enzymes (aspartate aminotransferase (SGOT) and alanine aminotransferase (SGPT)) and the severity of hepatic steatosis (*p* = 0.03, *p* = 0.05, and *p* = 0.02 for SGOT, SGPT, and liver steatosis, respectively) in individuals with NAFLD.	[54]
Badran et al. 2022	Ramadan fasting significantly improved body mass index, cholestrol, triglycerides, LDL, HDL (*p* ≤ 0.01), HbA1c, postprandial, HOMA-IR, and fibrosis markers (FIB-4 and APRI) (*p* < 0.01) in individuals with MASLD, especially in the initial phases and among those at risk for diabetes	[55]
Aliasghari et al. 2017	Ramadan fasting led to improvements in inflammatory cytokines, anthropometric indices, plasma insulin, and fasting plasma glucose in individuals with MASLD.	[56]
Alam et al. 2019	Fasting during Ramadan may enhance NAFLD by promoting weight loss. Weight reduction has been shown to enhance all aspects of histological activity in patients with nonalcoholic steatohepatitis in subjects with NASH.	[57]
Ahmed and Ahmed 2024	Ramadan fasting decreased body weight and produced improvements in lipid profile, fasting blood glucose, anthropometric indices, plasma insulin, and inflammatory cytokines. Ramadan fasting improved MASLD risk factors and might improve MASLD through reduction in body weight.	[51]
Lin et al. 2024	Fasting throughout Ramadan produced improvements in body weight, and composition, cardiometabolic risk factors, glucose levels, inflammation markers, and liver parameters.	[20]

**Table 2:** Effects of Ramadan fasting on heart failure and liver cirrhosis.

Effects of Ramadan Fasting	
Heart Failure	Liver Cirrhosis
Ramadan fasting significantly reduced the incidence of signs and symptoms of volume overload in comparison to those hospitalized in other months. The incidence of atrial arrhythmias was notably less frequent, and cholesterol serum levels were significantly reduced during Ramadan. Ramadan fasting improved the symptoms and quality life of patients with chronic heart failure. There was no significant difference in the number of hospitalizations for congestive heart failure during Ramadan fasting compared to during non-fasting months. Heart failure patients should be encouraged to fast during Ramadan.	Ramadan fasting resulted in significant reductions in anthropometric measurements in patients with cirrhosis. Ramadan fasting increased ascitic fluid production, new ascites, elevated plasma bilirubin levels post-Ramadan and frequently experienced hepatic encephalopathy and abrupt upper gastrointestinal hemorrhage. Patients with cirrhosis classified as Child−Pugh class B and C should refrain from fasting throughout Ramadan, although those classified as Child−Pugh class A may fast if they adhere to specific precautions.

## Data Availability

No data were used to support the findings of this study.
